# Different Strategies for Photosynthetic Regulation under Fluctuating Light in Two Sympatric *Paphiopedilum* Species

**DOI:** 10.3390/cells10061451

**Published:** 2021-06-10

**Authors:** Jing-Qiu Feng, Wei Huang, Ji-Hua Wang, Shi-Bao Zhang

**Affiliations:** 1 Key Laboratory of Economic Plants and Biotechnology, Yunnan Key Laboratory for Wild Plant Resources, Kunming Institute of Botany, Chinese Academy of Sciences, Kunming 650201, China; fengjingqiu@mail.kib.ac.cn (J.-Q.F.); huangwei@mail.kib.ac.cn (W.H.); 2 University of Chinese Academy of Sciences, Beijing 100049, China; 3 Flower Research Institute of Yunnan Academy of Agricultural Sciences, Kunming 650205, China

**Keywords:** cyclic electron flow, fluctuating light, *Paphiopedilum*, photosystem I, water-water cycle

## Abstract

Fluctuating light can cause selective photoinhibition of photosystem I (PSI) in angiosperms. Cyclic electron flow (CEF) around PSI and electron flux from water via the electron transport chain to oxygen (the water-water cycle) play important roles in coping with fluctuating light in angiosperms. However, it is unclear whether plant species in the same genus employ the same strategy to cope with fluctuating light. To answer this question, we measured P700 redox kinetics and chlorophyll fluorescence under fluctuating light in two *Paphiopedilum* (*P.*) Pftzer (Orchidaceae) species, *P. dianthum* and *P. micranthum*. After transition from dark to high light, *P. dianthum* displayed a rapid re-oxidation of P700, while *P. micranthum* displayed an over-reduction of P700. Furthermore, the rapid re-oxidation of P700 in *P. dianthum* was not observed when measured under anaerobic conditions. These results indicated that photo-reduction of O_2_ mediated by the water-water cycle was functional in *P. dianthum* but not in *P. micranthum*. Within the first few seconds after an abrupt transition from low to high light, PSI was highly oxidized in *P. dianthum* but was highly reduced in *P. micranthum*, indicating that the different responses of PSI to fluctuating light between *P. micranthum* and *P. dianthum* was attributed to the water-water cycle. In *P. micranthum*, the lack of the water-water cycle was partially compensated for by an enhancement of CEF. Taken together, *P. dianthum* and *P. micranthum* employed different strategies to cope with the abrupt change of light intensity, indicating the diversity of strategies for photosynthetic acclimation to fluctuating light in these two closely related orchid species.

## 1. Introduction

Photosynthesis is a vital path to energy conversion in photosynthetic organisms. Plants use light energy to generate reduced nicotinamide adenosine dinucleotide phosphate (NADPH) and adenosine triphosphate (ATP), which are utilized in the Calvin-Benson cycle and photorespiration. In linear electron flow (LEF), electrons from photosystem II (PSII) are transported to plastoquinone, cytochrome (Cyt) *b*_6_*f* complex, plastocyanin, and ultimately to photosystem I (PSI), reducing NADP^+^. During this electron transport, proton motive force is formed to generate ATP through chloroplast ATP synthase, and the ATP/NADPH production ratio produced by LEF is thought to be 1.29 [1,2]. However, the ATP/NADPH ratio required by primary metabolism is approximately 1.5 [3]. Therefore, plants need a flexible mechanism to increase the ATP/NADPH production ratio, which can be accomplished by the activation of cyclic electron flow (CEF) [4,5]. During CEF, electrons from ferredoxin are transferred to plastoquinone, producing ΔpH without reducing NADP^+^. The CEF-dependent formation of ΔpH not only provides additional ATP but also facilitates photoprotection via non-photochemical quenching (NPQ) induction and photosynthetic control at the Cyt *b*_6_*f* complex [5,6].

Under natural conditions, light conditions can strongly fluctuate at the timescales of seconds or minutes [7,8]. Sudden changes in light intensity can influence the growth of plants. Usually, the photosynthetic rate rapidly reduces upon an abrupt transition from high to low light [9,10,11,12]. Meanwhile, there are no excess electrons produced in PSII, preventing the over-reduction of PSI. However, light absorption and electron transfer from PSII to PSI rapidly increase with a sudden increase in light intensity [13,14,15]. Meanwhile, the Calvin-Benson cycle responds slowly, and thus the excited states cannot be immediately consumed by primary metabolism [13,16,17,18]. Under such conditions, the over-reduction of PSI induces the production of reactive oxygen species within PSI and thus causes PSI photoinhibition [19,20]. The CO_2_ and photoprotection are strongly limited by the occurrence of PSI photoinhibition, thus impairing plant growth [17,21,22,23]. Therefore, it is of great significance to understand the response of photosynthetic organisms to fluctuating light.

For early evolutionary organisms such as cyanobacteria, alga, ferns, and gymnosperms, they can rapidly consume excess electrons under fluctuating light by photoreduction of O_2_, depending on flavodiiron proteins [24,25,26,27]. For angiosperms, however, they use other pathways to cope with fluctuating light due to their lack of flavodiiron proteins [8,26,28,29,30,31,32]. Nevertheless, the strategy to cope with fluctuating light largely differs among angiosperms. In *Arabidopsis thaliana*, *Nicotiana tabacum*, and *Oryza sativa*, CEF is employed to protect PSI under fluctuating light [17,29,31,33]. In the shade-grown species *Paris polyphylla*, the downregulation of PSII activity diminishes excess electrons to PSI and thus prevents PSI photoinhibition under fluctuating light [34]. By comparison, *Camellia* species and *Bryophyllum pinnatum* use the water-water cycle (electrons originating from water splitting in PSII are ultimately used to reduce O_2_ to water) to consume the excess electrons in PSI, which prevents the over-reduction of PSI and thus avoids the photoinhibition of PSI under fluctuating light [30,34,35,36]. However, at present, it is unclear whether the strategy for coping with an abrupt increase from low to high light in angiosperms is consistent in closely related species.

The Orchidaceae family is one of the largest families of angiosperms with diverse life forms, life histories, habitats, and ecological characteristics [37,38]. A previous study has shown that an epiphytic orchid, *Dendrobium officinale*, uses the water-water cycle to consume excess electrons in PSI after an abrupt transition from low to high light [39]. However, in the terrestrial orchid *Bletilla striata*, the CEF around PSI is activated upon an abrupt transition from low to high light, which alleviates the over-reduction of PSI and prevents uncontrolled PSI photoinhibition [15]. These studies indicate that the strategy for coping with fluctuating light largely differs among orchids in different genera. However, the strategies used by orchids from the same genus to cope with fluctuating light is poorly understood.

Members of the genus *Paphiopedilum* (*P.*) Pftzer (Orchidaceae) are world-famous ornamental orchids because of their unique flower shapes, colors, and long flower lifespans. However, due to illegal collection and habitat destruction, the number of wild populations and individuals of *Paphiopedilum* have been drastically decreased [40]. Therefore, all known *Paphiopedilum* species are strictly protected and listed in the Convention on International Trade in Endangered Species of Wild Fauna and Flora. Previous studies have supplied ample data for the conservation of *Paphiopedilum*, such as their reproductive ecology and ex situ seed baiting [41,42]. Recently, reintroduction is considered an effective way to protect *Paphiopedilum* plants, but there remain many challenges in employing this strategy [43]. For example, orchid mycorrhizal fungi are severely lacking in situ [44]. Furthermore, the study of ecophysiological performance in *Paphiopedilum* species, especially photosynthesis, is still lacking.

In this study, the chlorophyll florescence and PSI redox state were measured under fluctuating light in two sympatric *Paphiopedilum* species, *P. dianthum* and *P. micranthum*. The aims of the study were to understand the strategy used to cope with fluctuating light in the two species, and to explore whether the closely related species employ the same photosynthetic strategy to cope with fluctuating light.

## 2. Materials and Methods

### 2.1. Plant Materials

In this study, *P. dianthum* and *P. micranthum* plants were used for the experiments. These two species mainly occur in limestone or mountainous forests of tropical and subtropical zones from north Vietnam to Guizhou Province, China. The plants were cultivated in the greenhouse of the Kunming Institute of Botany, Chinese Academy of Science (102°41′ E, 25°01′ N) (40% sunlight, 50–70% relative humidity). All plants were planted in porous plastic pots (10 cm × 15 cm) filled with bark and humus (7/3, *v*/*v*). At the greenhouse site, the maximum light intensity of sunlight is close to 800 μmol photons m^−2^ s^−1^ at midday. To ensure healthy growth of the plants, all plants were watered once per week and fertilized with controlled release fertilizer (Osmocote, nitrogen: phosphate: potash = 14:14:14, Geldermalsen, The Netherlands) at a rate of approximately 0.5 g per pot every month. Five fully expanded mature leaves per species from five individuals were used for photosynthetic measurements.

### 2.2. Chlorophyll Fluorescence and P700 Measurements

We used a Dual PAM-100 (Heinz Walz, Effeltrich, Germany) to measure PSI and PSII parameters. After dark adaptation for 15 min, the maximum change in P700 and the maximum fluorescence were recorded with a saturating pulse. Subsequently, the mature leaves were illuminated under fluctuating light alternating between 59 (3 min) and 1455 μmol photons m^−2^ s^−1^ (1 min) for 32 min. PSI and PSII parameters were recorded under this fluctuating light condition.

The P700^+^ signals (*P*) can vary between a minimum (P700 fully reduced) and a maximum level (P700 fully oxidized). The *P_m_*′ was determined by using a saturation pulse (300 milliseconds and 20,000 μmol photons m^−2^ s^−1^) after pre-illumination with actinic light for 10 s. The *P_m_* was similarly obtained, except that far-red light was used instead of actinic light. Afterwards, the quantum yield of PSI photochemistry (Y(I)), the quantum yield of PSI non-photochemical energy dissipation due to donor side limitation (Y(ND)), and the quantum yield of non-photochemical energy dissipation due to acceptor side limitation (Y(NA)) were calculated with the following formulas: Y(I) = (*P_m_*′ − *P*)/*P_m_*, Y(ND) = *P*/*P_m_* and Y(NA) = (*P_m_* − *P_m_*′)/*P_m_*.

PSII parameters were calculated as follows [45,46]: Y(II) = (*F_m_*′ − *F_s_*)/*F_m_*′, Y(NO) = *F_s_*/*F_m_*, and NPQ = (*F_m_* − *F_m_*′)/*F_m_*′. Y(II) was the effective quantum yield; Y(NO) was the quantum yield of non-regulated energy dissipation in PSII; and NPQ was the non-photochemical quenching in PSII. *F_s_* was the steady state after light adaptation. *F_m_* and *F_m_*′ represented the maximum fluorescence after dark and light adaptation, respectively. *F_m_* was recorded after dark-adaptation for 15 min. Photosynthetic electron flow through PSI and PSII (ETRI and ETRII, respectively) was calculated as follows: ETRI = PPFD × Y(I) × 0.84 × 0.5, ETRII = PPFD × Y(II) × 0.84 × 0.5, where 0.84 represents the leaf absorbance and 0.5 is the proportion of absorbed light energy allocated to PSI or PSII, and PPFD represents the photosynthetic photon flux density. The value of CEF around PSI was calculated according to the formula: CEF = ETRI − ETRII.

### 2.3. Redox Kinetics upon Dark-To-Light Transition

A Dual-PAM 100 (Heinz Walz, Effeltrich, Germany) was used to measure the redox kinetics of P700 upon dark-to-light transition. After the mature leaves were adapted to dark conditions for at least 1 h, they were suddenly exposed to actinic light (1455 μmol photons m^−2^ s^−1^) and the redox changes in P700 were measured over 16 s. To measure P700 under anaerobic conditions, the detached leaves were induced in nitrogen for at least 1 h.

### 2.4. Statistical Analysis

Statistical analysis was conducted using SPSS 20.0 (SPSS Inc., Chicago, IL, USA). All data were calculated based on five independent experiments. The difference between *P. dianthum* and *P. micranthum* was analyzed using the *t*-tests of independent samples.

## 3. Results

### 3.1. Response of PSI Parameters to Fluctuating Light

**Figure 1:**
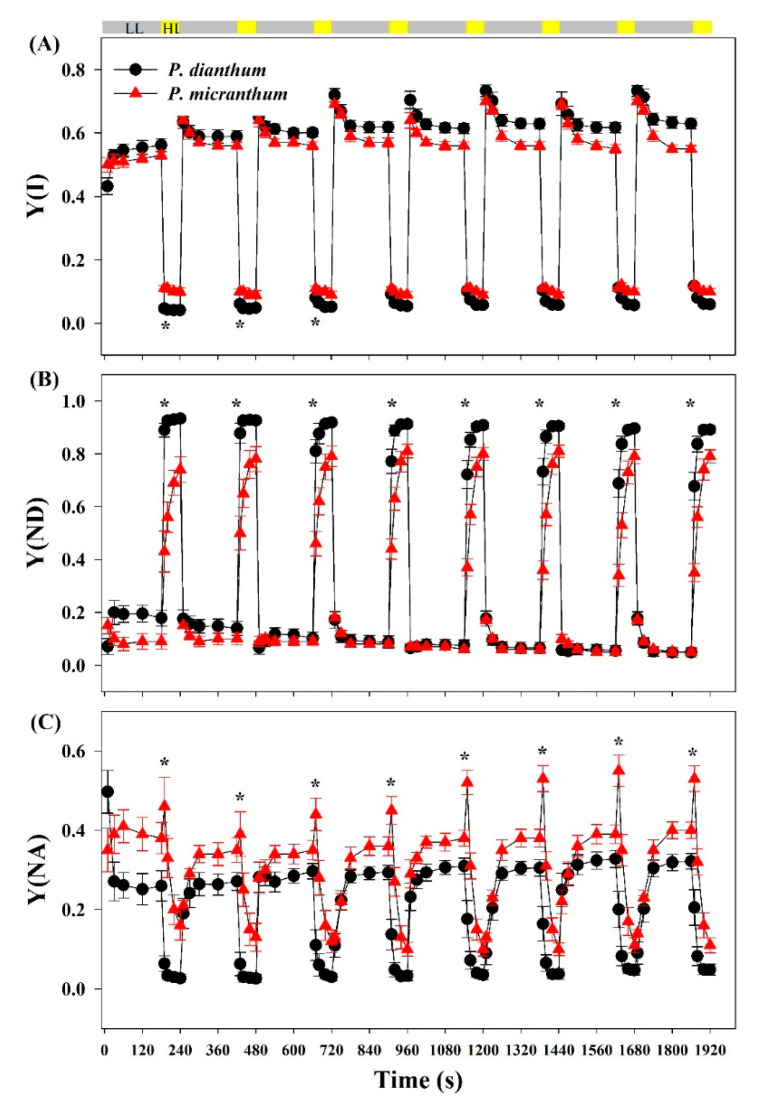
PSI parameters of *Paphiopedilum dianthum* and *P. micranthum* under fluctuating light. Y(I), effective quantum yield of PSI (**A**); Y(ND), PSI donor side limitation (**B**); Y(NA), PSI accept side limitation (**C**). Each data point represents the mean ± SE for five measurements from five individual plants. LL, low light; HL, high light. Asterisks indicate significant differences between *P. dianthum* and *P. micranthum* with the *t*-tests of independent samples.

After an abrupt transition from low to high light, the values of Y(I) in *P. micranthum* were significantly higher than those in *P. dianthum* in the first three cycles of low-high light (Figure 1A). The values of Y(ND) in *P. dianthum* were always significantly higher than those in *P. micranthum* after an abrupt transition from low to high light, indicating the stronger oxidation of PSI in *P. dianthum* (Figure 1B). The values of Y(NA) in *P. micranthum* were always significantly higher than those in *P. dianthum* in the high light phases under fluctuating light, indicating the strong acceptor side limitation in *P. micranthum* (Figure 1C). Surprisingly, the value of Y(NA) in *P. micranthum* significantly increased within the first 10 s after an abrupt increase in light intensity, while *P. dianthum* had an obviously opposite change. These results indicated that these two species showed different PSI performances under fluctuating light. The values of Y(NA) of the two species reduced to stable values within the first 40 s after a sudden transition from low to high light.

### 3.2. Response of PSII Parameters to Fluctuating Light

**Figure 2:**
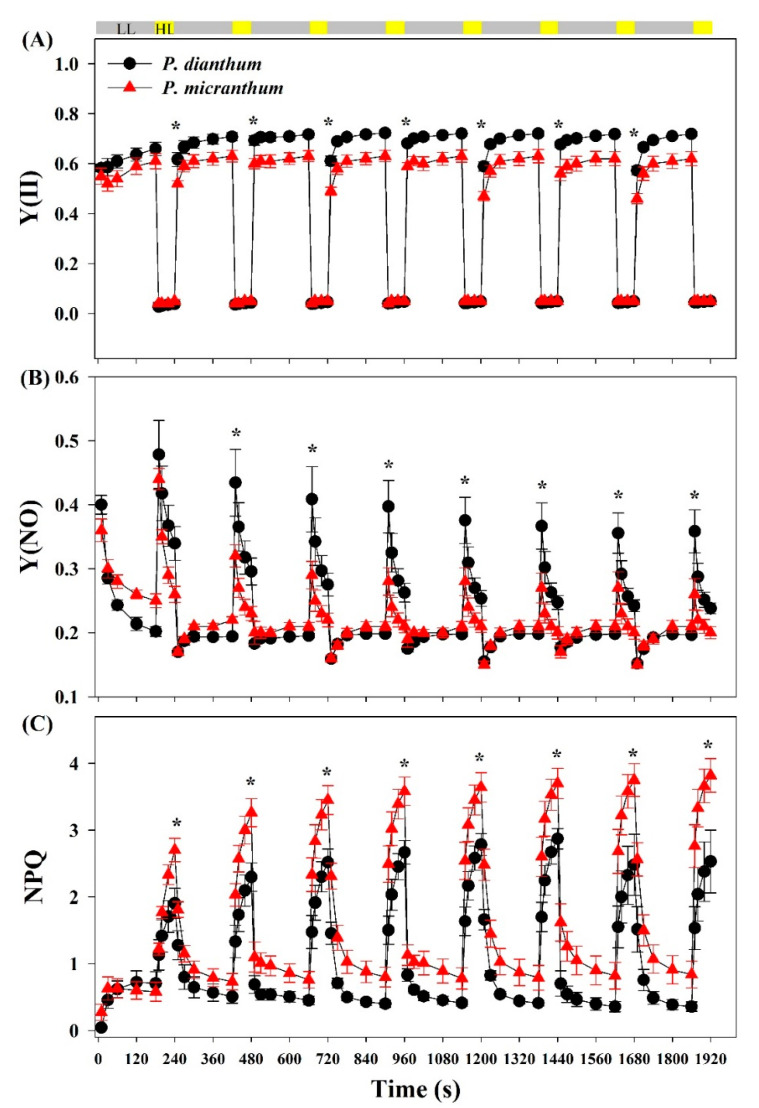
PSII parameters of *Paphiopedilum dianthum* and *P. micranthum* under fluctuating light. Y(II), effective quantum yield of photosystem II (**A**); Y(NO), quantum yield of non-regulated energy dissipation in PSII (**B**); NPQ, non-photochemical quenching (**C**). Each data point represents the mean ± SE for five measurements from five individual plants. LL, low light; HL, high light. Asterisks indicate significant differences between *P. dianthum* and *P. micranthum* with the t-tests of independent samples.

The mature leaves of *P. dianthum* and *P. micranthum* were exposed to fluctuating light with cycles of low-high light (59/1455 μmol photons m^−2^ s^−1^) after dark adaptation for at least 15 min. Compared with *P. micranthum*, the leaves of *P. dianthum* showed significantly higher Y(II) at low light, but there was no significant difference at high light (Figure 2A). The values of Y(NO) under low light were lower in *P. dianthum* than in *P. micranthum*, while the results were opposite under high light (Figure 2B). *P. dianthum* and *P. micranthum* showed similar values for NPQ during the first cycle of low-high light, but the values for NPQ under high light in *P. dianthum* were significantly lower than those in *P. micranthum* during the subsequent cycles (Figure 2C).

### 3.3. Response of Photosynthetic Electron Transport to Fluctuating Light

**Figure 3:**
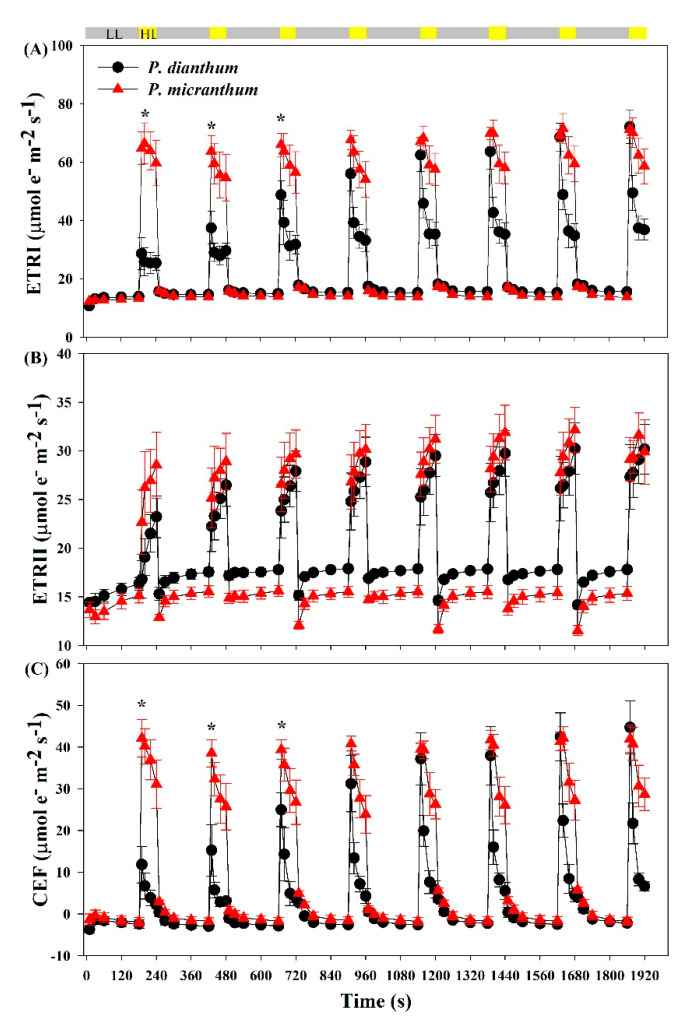
Photosynthetic electron transport of *Paphiopedilum dianthum* and *P. micranthum* under fluctuating light. ETRI, rate of photosynthetic electron flow in PSI (**A**); ETRII, rate of photosynthetic electron flow in PSII (**B**); CEF, cyclic electron flow around PSI (**C**). Each data point represents the mean ± SE for five measurements from five individual plants. LL, low light; HL, high light. Asterisks indicate significant differences between *P. dianthum* and *P. micranthum* with the t-tests of independent samples.

Fluctuating light obviously affected the photosynthetic electron flow in the two species. Within the first 10 s, the ETRI rapidly increased to a peak and then reduced to a stable value within 40 s after an abrupt transition from low to high light (Figure 3A). Within the initial three cycles of low-high light, the values of the ETRI in high light phases in *P. micranthum* were significantly higher than that in *P. dianthum*. The value of the ETRI under the steady state was significantly higher in *P. micranthum* than that in *P. dianthum* (Figure 3A). After an abrupt transition from low to high light, the ETRI gradually increased with the increasing cycles of fluctuating light in *P. dianthum*, while the ETRI reached the peak value at the first round in *P. micranthum*. The ETRII increased more slowly in *P. dianthum* than that in *P. micranthum* within the first two cycles after an abrupt transition from low to high light (Figure 3B), and *P. dianthum* showed a higher ETRII than that in *P. micranthum* under low light. Upon an abrupt transition from low to high light, the value of ETRI—ETRII first increased to a peak and then rapidly reduced to the steady state, suggesting the transient stimulation of CEF in both species (Figure 3C).

### 3.4. Correlation between PSI Redox State and CEF Activation

**Figure 4:**
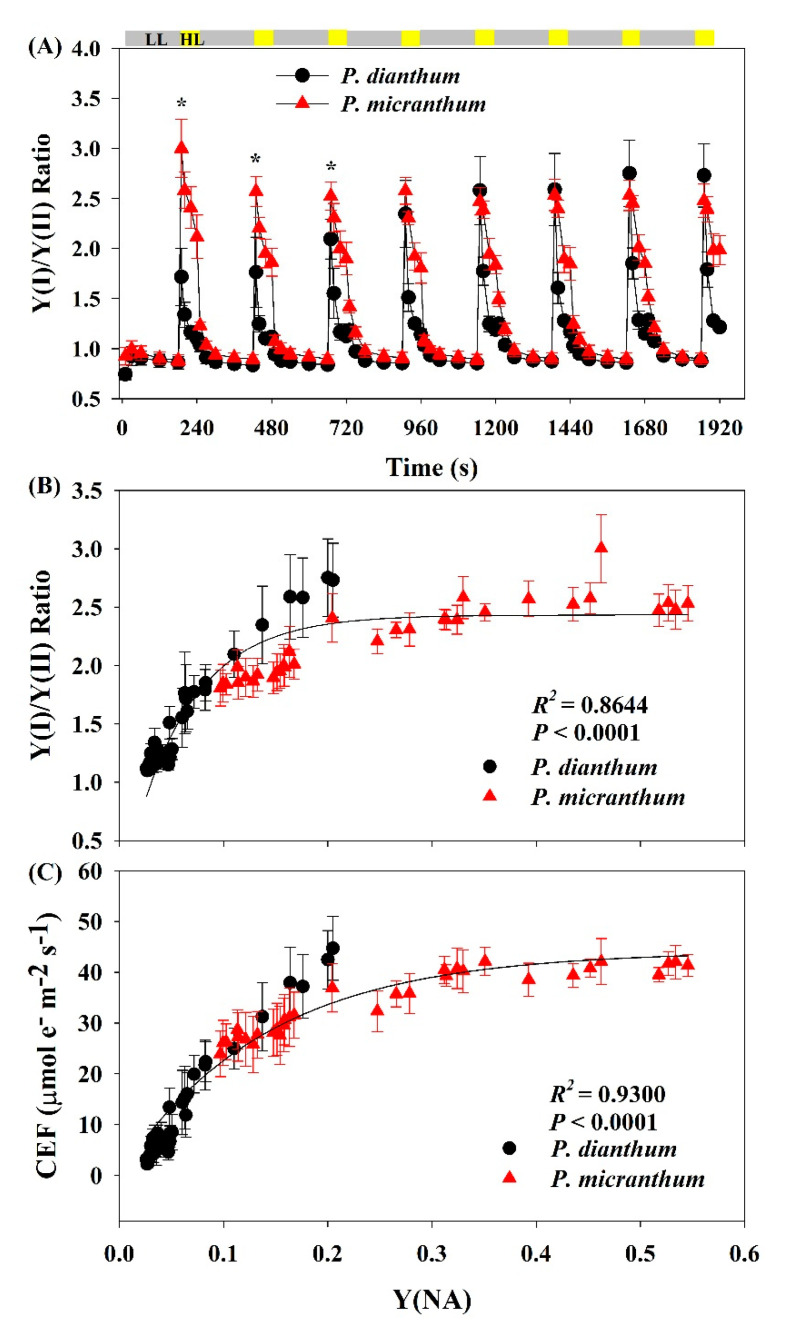
Changes in the Y(I)/Y(II) ratio under fluctuating light alternating between 59 and 1455 μmol photons m^−2^ s^−1^ for leaves of *Paphiopedilum dianthum* and *P. micranthum* (**A**). Relationship between Y(I)/Y(II) ratio (**B**), CEF (**C**), and Y(NA) after transition from 59 to 1455 μmol photons m^−2^ s^−1^. Y(I), effective quantum yield of PSI; Y(II), effective quantum yield of photosystem II; CEF, cyclic electron flow around PSI. Each bar represents the mean ± SE for five measurements from five individual plants. LL, low light; HL, high light. Asterisks indicate significant differences between *P. dianthum* and *P. micranthum* with the t-tests of independent samples.

Under constant low light, the values of the Y(I)/Y(II) ratio of *P. dianthum* and *P. micranthum* were similar (Figure 4A). The values of the Y(I)/Y(II) ratio in the two plants rapidly increased upon an abrupt transition from low to high light, indicating that the CEF within the first 10 s was stimulated in both species after an abrupt transition from low to high light. However, *P. micranthum* showed a much higher CEF stimulation compared to *P. dianthum* after the light intensity increased within the first 10 s. In addition, we found a significant relationship between the CEF activation and PSI redox state, suggesting that the over-reduction of PSI acted as an important signal for activation of CEF (Figure 4B,C).

### 3.5. Redox Kinetics upon Dark-Light Transition

**Figure 5:**
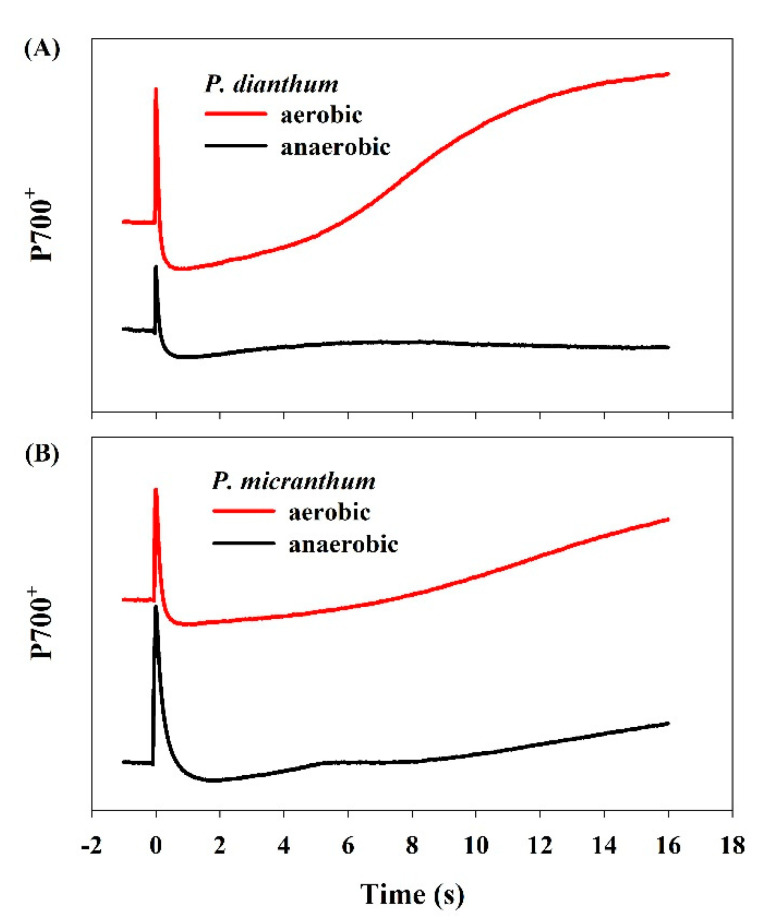
The redox changes of P700 upon dark-to-light transition in *Paphiopedilum dianthum* (**A**) and *P. micranthum* (**B**) under aerobic and anaerobic conditions. Each line represents the mean for five measurements from five individual plants. The red line and the black line are the measured values of P700^+^ under aerobic and anaerobic conditions, respectively.

To examine the alternative electron flow, the P700 redox kinetics upon the illumination of dark-adapted leaves to actinic light (1455 μmol photons m^−2^ s^−1^) were measured. Actinic light induced the initial peak of P700 oxidation, which was followed by its reduction and re-oxidation in *P. dianthum* (Figure 5). However, such rapid re-oxidation of P700 in *P. dianthum* was obviously missing when measured under anaerobic conditions. Similarly, the rapid re-oxidation of P700 was clearly missing in *P. micranthum*, even under aerobic conditions. These results indicated that a significant electron flow through the water-water cycle existed in *P. dianthum*, but not in *P. micranthum*.

## 4. Discussion

Many studies have documented that fluctuating light can lead to selective photoinhibition of PSI, which restricts photosynthetic CO_2_ assimilation and impairs the growth of plants [17,23,47,48,49]. To protect PSI under a sudden transition of light intensity, angiosperms can use several alternative electron flows to regulate the PSI redox state after an abrupt transition from low to high light, such as the CEF and the water-water cycle [17,20,33,35,50]. The CEF is considered the main way for angiosperms to flourish under fluctuating light. In addition, compared with the CEF, the water-water cycle is more efficient in protecting PSI under fluctuating light [30]. However, the activity of the water-water cycle is species specific among angiosperms. The water-water cycle significantly facilitates photoprotection for PSI under fluctuating light in the epiphytic orchid *D. officinale*, but does not function in another orchid, *B. striata* [39], indicating that diverse strategies for coping with fluctuating light exist within closely related species.

In this study, we examined the PSI and PSII parameters in response to fluctuating light in two closely related *Paphiopedilum* species, *P. dianthum* and *P. micranthum*. Our results indicated, after an abrupt transition from low to high light, that *P. micranthum* showed an over-reduction of PSI within the first 10 s. This phenomenon is consistent with the findings in *A. thaliana*, *N. tabacum*, and *O. sativa* [20,28,29,51]. However, such over-reduction of PSI was clearly missing in *P. dianthum*, which was similar to the phenomenon in species with significant activity of the water-water cycle, such as *B. pinnatum*, *Camellia* species, and *D. officinale* [35,36,39]. These findings indicated that the response of PSI to fluctuating light in angiosperms significantly differed among species, even among species in the same genus.

The donor and acceptor side regulation determine the redox state of PSI under fluctuating light. In donor-side regulation, a high level of ΔpH downregulates the plastoquinone oxidation at the Cyt *b_6_*/*f* complex and thus controls the electron flow from PSII to PSI [33,52,53]. Once the formation of ΔpH is inhibited under high light, the excess electron flow to PSI resulting from an insufficient ΔpH causes the over-reduction of PSI electron carriers and PSI photoinhibition. Interestingly, within the first second after an abrupt transition from low to high light, plants cannot produce a sufficient ΔpH, which causes the PSI photoinhibition under fluctuating light in *A. thaliana*, *N. tabacum*, and *O. sativa* [20,28,29,51]. The values of NPQ in *P. micranthum* and *P. dianthum* did not rise to the maximum level within 40 s of the abrupt transition from low to high light, indicating relatively low ΔpH during this period in both species. Therefore, in *P. micranthum*, the transient over-reduction of PSI under fluctuating light was also linked to the slow kinetics of ΔpH formation. In contrast, after an abrupt transition from low to high light within 10 s, *P. dianthum* showed a rapid oxidation of PSI electron carriers, which was caused by other factors rather than the ΔpH formation. Thus, the difference in PSI response to fluctuating light could not be explained by the donor side regulation.

In acceptor side regulation, outflow of electrons from PSI to downstream electron acceptors consumes a significant fraction of reducing power in PSI and thus facilitates the rapid oxidation of PSI electron carriers. In non-angiosperms, a fast oxidation of PSI was regulated by the photoreduction of O_2_ mediated by flavodiiron proteins upon any increase in light intensity [13,24,54]. By comparison, in flowering plants the oxidation of PSI is attributed to LEF and the water-water cycle [30]. In LEF, the electron transfer from PSI to NADP^+^ is largely affected by the NADP^+^/NADPH ratio that in turn is determined by the operation of CO_2_ fixation. Upon an abrupt transition from low to high light, because of the slow kinetics of stomata and activation of related enzymes, the full activation of the Calvin cycle requires several minutes [17,55]. Consequently, the electron transfer from PSI to NADP^+^ is usually restricted under fluctuating light. Once the excess reducing power in PSI cannot be consumed by the water-water cycle, PSI might be over-reduced under fluctuating light, which was shown to be the case for *P. micranthum* in the present study. However, if the water-water cycle is operational, the excess excited states in PSI are consumed by the Mehler reaction, and the over-reduction of PSI could be prevented. This conclusion is supported by the photosynthetic regulation in some groups of angiosperms, such as *B. pinnatum*, *Camellia* species, and *D. officinale* [35,36,39]. In the present study, we found that *P. dianthum* showed a rapid re-oxidation of P700 upon transition from dark to light, and such rapid re-oxidation clearly disappeared under anaerobic conditions. Thus, the water-water cycle was operational in *P. dianthum*, which led to the abrupt oxidation of PSI electron carriers upon any transitions from low to high light. Consequently, the water-water cycle was responsible for the diversity of strategies for photosynthetic regulation in closely related taxon under fluctuating light.

In this study, the contribution of the water-water cycle to total photosynthetic electron flow was neglected in *P. micranthum*. However, it can be partially compensated by the transient activation of CEF under fluctuating light. As shown in Figure 4, the stimulation of CEF under fluctuating light was largely linked to the PSI redox state. Within the first 10 s after an abrupt increase in light intensity, the severe over-reduction of PSI occurred with the high stimulation of CEF. Under such conditions, CEF contributed to the major part of the total photosynthetic electron flow. This activation of CEF directly helps the abrupt formation of ΔpH, which prevents uncontrolled photoinhibition of PSI through two different mechanisms: one is linked to ΔpH-dependent photosynthetic control at the Cyt *b_6_*/*f* complex, and the other is linked to the activation of CO_2_ fixation at the step of ATP supplement [8]. A recent study reported that overproduction of PGR5 contributed to an enhanced electron sink downstream of PSI under fluctuating light in a C_4_ plant, *Flaveria bidentis* [56]. This implies that CEF-dependent ATP synthesis favors the activation of primary metabolism, which in turn facilitates electron flow from PSI to NADP^+^. Thus, the transient stimulation of CEF played an important role in photoprotection under fluctuating light in both of the *Paphiopedilum* species assessed in the present study, especially in *P. micranthum*.

Under natural conditions, *P. dianthum* and *P. micranthum* can occur in the same areas throughout China, but the former species is mainly distributed in northern parts of the tropical zone, while the latter is mainly distributed in southern parts of the subtropical zone [40]. *P. dianthum* mainly grows in the edge of forests while *P. micranthum* mainly grows under the forest canopy [57]. Compared with understory plants, the forest edge plants are usually exposed to more intense fluctuating light conditions. In our study, *P. dianthum* used the water-water cycle to cope with fluctuating light while CEF was used by *P. micranthum* to cope with fluctuating light. These findings are consistent with the strategies for coping with fluctuating light in other orchid species, such as *D. officinale* and *B. striata* [39]. Sun et al. [36] showed that the water-water cycle is used by 11 *Camellia* species, indicating that the strategy to cope with fluctuating light might be consistent among species in the same genus. In our study, however, the two members of *Paphiopedilum*, *P. dianthum* and *P. micranthum*, used different strategies to cope with fluctuating light. These results showed that the photosynthetic regulation of plants under fluctuating light was affected by their native habitats rather than their phylogenetic relationship.

## 5. Conclusions

In this study, the photosynthetic regulation of two closely related *Paphiopedilum* species were compared under fluctuating light. Upon an abrupt transition from low to high light, *P. dianthum* displayed a rapid oxidation of PSI, while *P. micranthum* demonstrated an over-reduction of PSI. Moreover, our findings indicated that the water-water cycle was used for the rapid oxidation of PSI in *P. dianthum* rather than CEF. However, CEF was highly activated in *P. micranthum* to offset the weak function of the water-water cycle. Thus, during fluctuating light, a variety of strategies are employed to avoid PSI photoinhibition among angiosperms, even among species in the same genus. The findings provide a new insight into the ecological adaptation of orchids.

## Data Availability

Not applicable.
